# A Hybrid Approach to Universal Intrusion Detection Systems for Automotive Security

**DOI:** 10.3390/s26051489

**Published:** 2026-02-27

**Authors:** Md Rezanur Islam, Mahdi Sahlabadi, Munkhdelgerekh Batzorig, Kangbin Yim

**Affiliations:** 1 Department of Software Convergence, Soonchunhyang University, Asan-si 31538, Republic of Korea; arupreza@sch.ac.kr; 2 Department of Information Security Engineering, Soonchunhyang University, Asan-si 31538, Republic of Korea; sahlabadi@sch.ac.kr (M.S.); munkh@sch.ac.kr (M.B.)

**Keywords:** CAN, IVN, wavelet, Pearson, IDS

## Abstract

Security measures are essential in the automotive industry to detect intrusions in-vehicle networks. However, developing a one-size-fits-all intrusion detection system (IDS) is challenging because each vehicle has a unique data profile. This is due to the complex and dynamic nature of the data generated by vehicles regarding their model, driving style, test environment, and firmware update. To address this issue, a universal IDS has been developed that can be applied to all types of vehicles without the need for customization. Unlike conventional IDSs, the universal IDS can adapt to data distribution shifts caused by changes in driving style, vehicle platform, or firmware updates. In this study, a new hybrid approach has been developed, combining Pearson correlation with deep learning techniques. This approach has been tested using data obtained from four distinct mechanical and electronic vehicles, including Tesla, Sonata, and two Kia models. The data has been combined into two frequency datasets, and wavelet transformation has been employed to convert them into the frequency domain, enhancing generalizability. Additionally, a statistical method based on independent rule-based systems using Pearson correlation has been utilized to improve system performance. The system has been compared with eight different IDSs, three of which utilize the universal approach, while the remaining five are based on conventional techniques. The accuracy of each system has been evaluated through benchmarking, and the results demonstrate that the hybrid system effectively detects intrusions in various vehicle models.

## 1. Introduction

Artificial intelligence is accelerating the shift toward connected, software-defined, and increasingly automated vehicles [1], but this same connectivity enlarges the attack surface of in-vehicle networks (IVNs), making intrusion detection a safety-critical requirement [2,3]. Modern vehicles comprise many ECUs, sensors, and communication links, yet CAN traffic is inherently non-portable: differences in network architecture and ECU composition produce vehicle-specific traffic profiles, and message generation further changes with driving conditions and driver behavior. As a result, IDS models trained on a single vehicle or a single collection context often fail to generalize, because they face both inter-vehicle and intra-vehicle distribution shifts [4,5,6]. This challenge is exacerbated by the limited availability of datasets that span diverse real-world conditions, which encourages evaluations without strict cross-validation and can overstate performance while hiding poor cross-vehicle robustness. Hence, the core problem is to develop an IVN IDS that remains reliable under heterogeneous real-world environments by explicitly accounting for distribution shift across vehicles and operating contexts.

IDSs primarily monitor controller area network (CAN) data because it is the main communication protocol within IVN [7]. IDSs face challenges when deployed across different CAN datasets [8]. CAN networks vary in function and design to meet specific vehicle needs and cater to the complexity of electronic and autonomous vehicles. For instance, Kia has three distinct CAN channels with electronic control unit (ECU) numbers of approximately 100, each responsible for different functions [9]. CAN networks handle crucial functions with B-CAN, C-CAN, and M-CAN to manage body, chassis, and multimedia systems, respectively. These networks vary in speed and priority to cater to specific vehicle needs. Although the Kia models share the same channel, their internal physical CAN ID and payload differ [6]. Similarly, the BMW K-CAN, V-CAN, and K2-CAN consist of different components according to the manufacturer’s internal functional design [10]. A significant difference between mechanical and electronic vehicles is their IVN architectures. This is because manufacturers offer various features to attract customers, and autonomous vehicles require many sensors to ensure safety [11]. For example, Tesla’s ECUs vary in number, with each version around four ECUs [12], and it has nine types of CAN channels [13]. CAN networks undoubtedly vary, as demonstrated by these industrial examples.

Conventional deep learning CAN IDS pipelines often learn from vehicle-dependent internal features such as CAN IDs and payload bytes. However, these structures differ across manufacturers and can change within the same vehicle after firmware updates, configuration changes, or behavioral shifts; moreover, the same CAN ID may encode driver- or context-dependent payload fields, making feature semantics unstable across domains. To reduce this brittleness, a UIDS should rely on vehicle-agnostic observables that remain comparable across platforms and operating conditions. Accordingly, we do not use CAN ID or payload as primary inputs and instead focus on statistical timing and traffic-volume signatures that are consistently available across vehicles. Despite the need for stronger IVN security, IDS deployment remains difficult because network architectures and data-generation patterns vary widely, motivating cross-domain approaches such as transfer learning [14] and federated learning [6]. Yet transfer learning can degrade performance when source and target domains share little common structure (negative transfer) [15], and federated learning introduces communication overhead and additional privacy and security concerns that limit scalability for high-rate vehicular data [16]. In contrast, the UIDS goal is to detect attacks across heterogeneous vehicles without per-platform retraining, improving interchangeability, reliability, and regulatory compliance [7]. However, existing universalization efforts remain limited in both capability coverage and evaluation breadth, motivating the approach and validation protocol proposed in this study.

Existing universalization directions still leave important gaps: methods that combine multi-resolution transforms with fixed, rule-based decision logic can be fragile when an attacker shifts the injection frequency, many CAN-IDS studies are not evaluated under strict cross-vehicle protocols so generalization to unseen vehicles remains unclear, and approaches that directly use CAN IDs and payload bytes often learn source-vehicle-specific patterns that cause overfitting and contradict universality. CNN–RNN hybrids add runtime overhead while still relying on vehicle-specific semantics. To address these limitations, we propose a frequency-robust universalization strategy based only on vehicle-agnostic timing and traffic-volume statistics, standardized via multi-resolution features, and coupled with deep representation learning plus correlation-based consistency checks; positioned as a pilot UIDS study to mitigate data-availability constraints, we report measurable gains under an explicit transfer protocol (train on low-frequency injections from two vehicles; test across three vehicles under higher-frequency injections), with the following contributions:

- Vehicle-agnostic universal input: A universal feature representation based solely on timing and traffic-volume statistics, avoiding direct dependence on CAN IDs and payload bytes.
- Cross-vehicle, cross-frequency protocol: An explicit evaluation protocol for transfer across vehicles and injection-frequency domain.
- Quantified generalization gains: The hybrid decision improves cross-vehicle low-frequency accuracy by ∼8–10 percentage points and improves cross-frequency transfer accuracy by up to ∼3–10 percentage points.

## 2. Related Works

The section on universal approaches examines the limitations of existing IDSs, highlighting their constraints. The feature selection section explores the adaptability challenges of conventional IDSs in practical situations. Finally, the data conversion section focuses on the concept of data generalization.

### 2.1. Universal Approached Intrusion Detection

To the best of our knowledge, only three studies [17,18,19] have been conducted on a universal approach. Firstly, Novikova et al. [17] proposed an unsupervised anomaly detection approach for the CAN bus, identifying consistent signals across nine vehicles grouped into 32 subgroups. However, practical implementation faces challenges due to the requirement for separate autoencoder models for each subgroup, especially in resource-constrained IVN [20,21]. Accuracy measurements like false positive rates were not provided to evaluate their IDS.

Secondly, Bozdal et al. introduced WINDS [18], a Wavelet-based intrusion detection system with a specific accuracy matrix, requiring lower computational resources compared to [17]. It aims to universally enhance vehicle security against specific CAN ID injections. WINDS is rule-based, employing data generation to extract features transformed into wavelet coefficients, demanding high computational power [22]. However, it may generate false alerts with attack frequency changes and lacks cross-validation [23].

Thirdly, Islam et al. utilized a combination of heatmaps from CAN ID sequences [19], time gaps, and hamming distances of CAN IDs to develop a UIDS from real vehicles. They applied various CNN architectures as IDS to find the best model. However, a concern is that the study was conducted on only one vehicle with only high-frequency injection. Therefore, further research is needed to validate the ability of the IDS to be manufacturer-independent and demonstrate UIDS capability.

In summary, Novikova et al. conducted a study on achieving UIDS capability [17]. They utilized diverse datasets from different vehicles, emphasizing the importance of multi-source data. In contrast, Bozdal et al and Islam et al. employed Continuous Wavelet Transform (CWT) and heatmap techniques [18,19], highlighting the significance of feature selection and data conversion. These methodologies enhance data generalization and standardization, ultimately improving adaptability and facilitating analysis across various applications in the vehicle network system. More details on this topic can be found in Section 2.2.

### 2.2. Feature Selection for Intrusion Detection

This section reviews conventional IDSs methodology and input features (CAN ID sequence-based, payload sequence-based, full data utilization-based, voltage signal-based, and hybrid-based detection) from the UIDS point of view. Numerous studies [2,24,25,26,27] have been done on IVN security, mainly on their applicability to data sources in vehicular environments.

Yu et al. introduced TCE-IDS [2], employing a time interval conditional entropy algorithm to analyze decimal message IDs and data blocks from the CAN network in real-time for cyberattack detection. However, it relies on predefined rules and lacks the generalizability of a UIDS, necessitating frequent customization for vehicle or firmware updates.

Xun et al. propose an approach focusing on voltage signals from the CAN bus [24], using FeatureBagging combined with a CNN for identifying physical layer attacks. While effective, it may be less proficient in detecting application layer attacks [28]. Implementing an IDS at the application layer offers context awareness and content inspection advantages. However, a UIDS may require assistance with voltage features due to diverse ECU hardware configurations.

Mansourian et al. propose a payload-based IDS that utilizes LSTM and ConvLSTM prediction models with a Gaussian Naïve Bayes classifier [25], achieving high precision in distinguishing attack-free from attack data. However, the approach faces challenges in reliably detecting fuzzing attacks, incurs non-trivial computational overhead on resource-constrained ECUs, and can be undermined by adversaries who inject packets to modify CAN IDs while preserving payload patterns.

Jedh et al. studied CAN ID sequence-based IDSs [26], utilizing Messages Sequence Graphs and various techniques, including cosine similarity, Pearson correlation, threshold-based methods, LSTM-RNNs, and Change Point Detection (CPD). While effective, the study’s limited dataset raises concerns about generalizability to diverse vehicles and driving conditions. Additionally, attackers manipulating payload while maintaining CAN ID consistency pose challenges for the IDS.

Lo et al. presented the HyDL-IDS [27], a hybrid CNN-LSTM-based intrusion detection system for IVN. While effective, it has limitations, including potential bottlenecks in LSTM’s information extraction and computational overhead, especially for ECUs with limited capacity. If the primary model fails, the entire system may fail. Another limitation is that the CAN ID and payload are standardized according to CAN DBC [29], requiring customization for each vehicle [30].

As a result, it is important to address the limitations of current IDSs. These systems operate only with specific data sources from particular vehicles and lack the ability to generalize data.

### 2.3. Data Generalization for Intrusion Detection

Wavelet transforms enhance data generalization by capturing both time and frequency information [31], enabling a multi-resolution view that isolates key features while reducing noise [22,32]. This transform allows for efficient dimensionality reduction and temporal localization, which improves pattern recognition in non-stationary data like signals or time series. By compressing data and retaining only essential features, wavelets help machine learning models focus on relevant patterns, leading to improved generalization and reduced overfitting.

CAN systems generate large amounts of data (Table 1) that are difficult to analyze due to their non-stationary nature. Frequency domain analysis is a helpful approach to manage this complexity and gain a better understanding of the data. The Fourier transform is a commonly used technique in frequency domain analysis, but it has the drawback of compromising frequency and time resolution, which is problematic when dealing with non-stationary data [33,34]. This compromise is a common challenge in extensive data analysis, especially with large-scale datasets. Wavelet analysis is a powerful tool for handling evolving data. Unlike the Fourier transform, wavelet analysis is effective in dealing with non-stationary data [32]. It uses a wavelet function to analyze data at multiple scales, allowing the identification of broad and fine-scale patterns. This approach provides valuable insights into the frequency and time characteristics of the data [35], making it particularly suitable for datasets with dynamic frequency patterns. Wavelet analysis is widely used in various domains, including image analysis, telecommunications, anomaly detection, and biomedical data analysis [36,37,38,39,40,41]. The wavelet transform builds upon the short-time Fourier transforms, offering high-frequency resolution at lower frequencies and high-time resolution at higher frequencies [42]. This characteristic overcomes the limitations of Fourier transforms, making it valuable in extensive data analysis.

On the other hand, Pearson correlation is a useful method for detecting cyberattacks as it can identify unusual network traffic patterns [26,43,44]. It measures the linear relationship between variables and effectively detects deviations from expected behavior in network data. Analyzing correlations between various network parameters can reveal anomalies and potentially malicious activities. The resilience to outliers and ability to capture positive and negative correlations make Pearson correlation a valuable tool for identifying subtle attack patterns in complex network environments.

Securing the IVN is challenging and time-consuming due to the unique data profiles of each vehicle. Conventional IDS solutions require customization for each vehicle type and struggle with firmware updates and configuration changes. A UIDS is needed to simplify security measures, eliminate the need for customization, and ensure robust protection against evolving data patterns and adversarial attacks. The solution incorporates advanced machine learning techniques and a two-stage verification process, providing a standardized and effective security solution for all types of vehicles.

## 3. Features Extraction and Data Preprocessing

### 3.1. Data Collection

Vehicle-related study requires data collection, and the On-Board Diagnostics II (OBD-II) system is a commonly used method; however, not all ECUs can be accessed via the OBD-II port, which limits the amount of data that can be collected. To overcome this limitation, researchers use the ECU Direct Approach (EDA) [45], which involves acquiring data through an internal gateway using line-tapping tools while accessing the vehicle’s CAN network through an integrated central unit (ICU) [45]. The PEAK CAN system is the interfacing device for collecting normal driving data through the EDA method, and all preprocessing, as well as model training/inference, was conducted on a workstation equipped with an Intel Core i9 CPU and an NVIDIA RTX A6000 GPU using Python 3.10 with CUDA 12.9. This study employed a testbed framework incorporating real-world data to address safety concerns for attack data collection, where attacks such as fuzzing, DoS, and replay were simulated in a controlled environment to generate attack data for analysis and as input for deep learning models.

### 3.2. Feature Extraction

**Figure 1:**
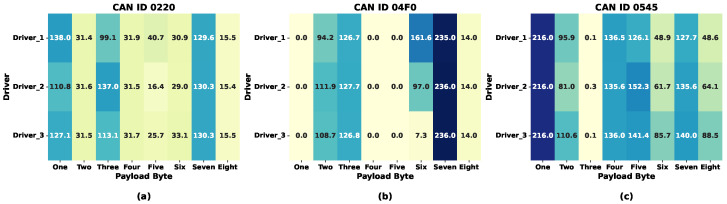
Heatmaps of the mean CAN payload byte values (Bytes 1–8) for three drivers. For each byte position, similar color intensity across drivers indicates that the mean payload value is stable (driver-invariant), whereas a visible color shift across drivers indicates driver-dependent changes in the mean payload value. (**a**) CAN ID 0×0220; (**b**) CAN ID 0×04F0; (**c**) CAN ID 0×0545.

This study analyzes diverse vehicle data to detect unauthorized data injections in vehicles. Four datasets of different vehicles were examined to identify common features that can help improve vehicle security. The results show differences in internal IDs and payload data among vehicles. The CAN DBC formation causes differences between vehicles of different manufacturers and models [29]. The internal data structure may change significantly following a firmware update or a change in driver behavior. Figure 1 presents driver-wise mean CAN payload heatmaps for three representative CAN IDs (0 × 0220, 0 × 04F0, and 0 × 0545). For each CAN ID, CAN frames were filtered by identifier, payload bytes were converted from hexadecimal to integer values, and mean byte values were computed per driver. Rows correspond to drivers, columns to payload bytes, and color intensity indicates the average payload magnitude using a unified color scale. While some bytes remain consistent across drivers, reflecting protocol-controlled fields, others exhibit clear driver-dependent variations. Notably, Bytes Three and Five in CAN ID 0 × 0220, Byte Six in CAN ID 0 × 04F0, and Bytes Five to Eight in CAN ID 0 × 0545 show systematic differences across drivers, suggesting that specific payload fields capture driver-dependent operational characteristics. As a result, conventional IDSs that directly use internal features such as CAN IDs and payloads face challenges in real-world performance. Therefore, this study avoids vehicle-specific internal features (e.g., CAN IDs and payloads) and instead analyzes vehicle-agnostic timing and traffic-volume statistics that exhibit stable periodic structure under attack-free conditions but become disrupted under message injection. In contrast, the proposed statistical features derived from attack-free traffic exhibit a stable periodic structure, which is disrupted when an attacker injects messages and perturbs the normal timing and volume patterns. To highlight these time–frequency irregularities and improve detectability, we apply a wavelet transform that represents the signals jointly in the time and frequency domains, making injection-induced deviations more separable for downstream detection. Therefore, this study avoids relying on such physical and internal features for attack detection and instead analyzes statistical features that exhibit common patterns indicative of unauthorized message injection.

**Figure 2:**
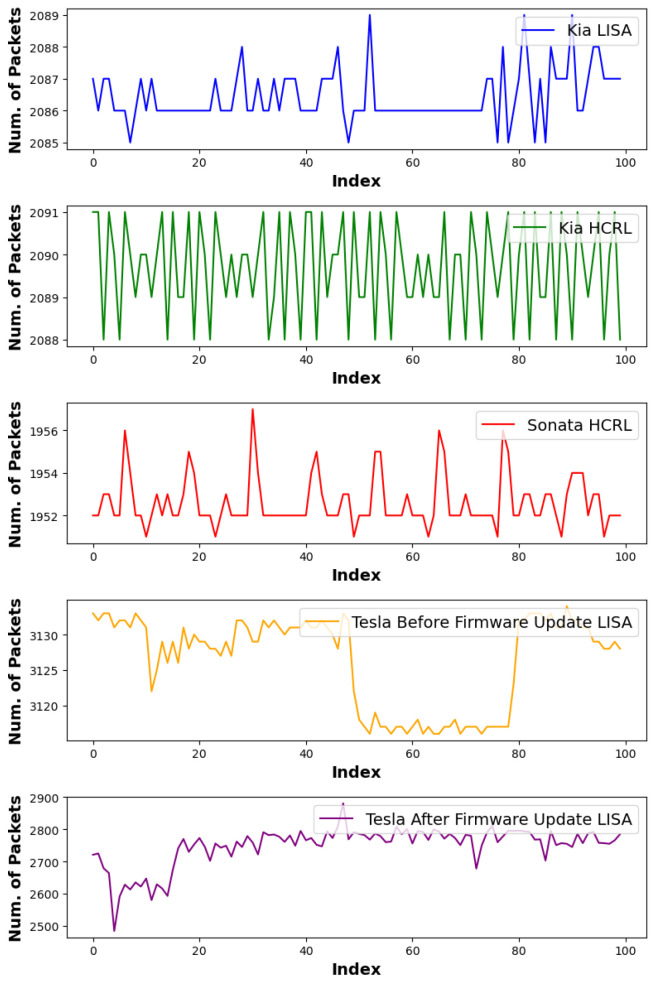
Data generation variability among vehicles.

Table 1 illustrates that Kia (LISA) and Kia (HCRL) exhibit similar statistical features despite sharing the same manufacturer and model. Although Sonata (HCRL) may have fewer CAN IDs, its data generation and average time gap metrics are consistent with those of other vehicles. Furthermore, the Tesla (LISA) autonomous vehicle generates data at the highest rate and with the shortest time intervals between messages, prioritizing safety and relying on numerous sensors. The amount of data generated by the Tesla vehicle varies depending on the driver’s activity or firmware updates, resulting in a noticeable discontinuity in the statistical features shown in Figure 2, where the data generation process of electronic vehicles fluctuates arbitrarily compared to that of mechanical vehicles.

### 3.3. Data Generalization

The primary objective of this study is to transform time-series CAN traffic statistics into the frequency domain to enhance cross-vehicle generalization for an IDS. Raw CAN logs are initially segmented into fixed 0.01-s intervals. For each segment, two fundamental statistical features are extracted: (i) the total number of packets and (ii) the average inter-arrival time gap. To capture high-resolution frequency components, we aggregate a window of 100 consecutive segments—equivalent to 1 s of statistical data—for subsequent transformation.

Given a discrete feature sequence x[n] derived from these metrics, the discrete wavelet transform (DWT) computes a multi-resolution representation using a two-channel analysis filter bank. For each decomposition level *j*, the approximation coefficients $c_{j+1}$ and detail coefficients $d_{j+1}$ are obtained by convolving the signal with a low-pass filter h[n] and a high-pass filter g[n], followed by dyadic downsampling:

(1) $$c_{j+1}\left[k\right]=\sum_{n}h\left[n-2k\right]\;c_{j}\left[n\right],$$

(2) $$d_{j+1}\left[k\right]=\sum_{n}g\left[n-2k\right]\;c_{j}\left[n\right].$$

In this formulation, h[·] and g[·] denote the scaling and wavelet filter coefficients, respectively. The factor 2k implements dyadic downsampling, which ensures computational efficiency while preserving the most informative subband content. As defined in Equations (1) and (2), this procedure decomposes the input sequence into constituent frequency components, facilitating the simultaneous analysis of coarse-scale trends and fine-grained fluctuations.

In this study, the Daubechies 8 (db8) wavelet and a 10-level DWT were utilized to analyze the CAN bus traffic. The selection of the db8 wavelet is justified by its eight vanishing moments, which provide an optimal equilibrium between spectral leakage suppression and the preservation of temporal localized transients. This characteristic is critical for CAN bus signals, where malicious injections typically manifest as subtle, high-frequency innovations superimposed on a periodic baseline. This specific decomposition depth ensures that the approximation coefficients ($A_{10}$) effectively encapsulate the fundamental network behavior, while the detail coefficients ($D_{1}$–$D_{10}$) provide sufficient frequency resolution to isolate low-volume, stealthy attacks that would remain obscured in shallower decompositions.

The transformation, executed via the pywt.wavedec function with symmetric boundary extension to minimize edge artifacts, yields the coefficient set $\left[cA_{10},cD_{10},\ldots ,cD_{1}\right]$. We discard the final approximation band $cA_{10}$ and retain the ten detail bands $\left\{cD_{1},\ldots ,cD_{10}\right\}$ as multi-resolution input features for the downstream ResNet-50 model. Although we refer to this as a frequency-domain conversion for convenience, the DWT more precisely provides a time–frequency (multi-resolution) representation rather than a pure frequency-domain mapping. In addition, while db8 is selected as a practical choice due to its smoothness and compact support properties, it is not universally optimal without task-specific comparison. Finally, under the standard DWT indexing convention, lower-order detail bands (e.g., $cD_{1}$) correspond to higher-frequency components, whereas higher-order detail bands (e.g., $cD_{10}$) capture lower-frequency variations; thus, periodic low-frequency behaviors are expected to be more pronounced in higher-order detail coefficients, while sharp transients manifest in lower-order detail coefficients. To accommodate differing coefficient lengths across levels and maintain uniform input dimensions for the deep learning architecture, a zero-padding technique is applied to the shorter subbands.

### 3.4. Pearson Correlation Analysis

In addition to the multi-resolution frequency features, this study utilizes the Pearson correlation coefficient (ρ) to characterize the linear dependency between the extracted statistical metrics. This coefficient serves as a diagnostic feature to monitor the consistency of the IVN data flow by quantifying the linear relationship between packet frequency and inter-arrival timing.

The Pearson correlation coefficient is defined as the ratio between the covariance of two variables and the product of their respective standard deviations:

(3) $$\rho_{X,Y}=\frac{\mathrm{cov}(X,Y)}{\sigma_{X}\sigma_{Y}},$$

where *X* represents the packet count (i.e., the total number of CAN frames within a segment) and *Y* represents the mean inter-arrival time gap. The term cov(X,Y) denotes the covariance, quantifying the extent to which the two metrics co-vary, while $\sigma_{X}$ and $\sigma_{Y}$ denote the standard deviations of *X* and *Y*, respectively.

By definition, ρ is constrained to the interval [−1,1], where ρ=1 indicates a positive linear relationship, ρ=−1 indicates a negative linear relationship, and ρ=0 implies the absence of linear correlation. Under normal driving conditions, the relationship between packet volume and inter-arrival timing remains relatively stable. In contrast, unauthorized data injections (e.g., DoS, fuzzing, replay) can induce a stronger negative dependency by abruptly increasing packet volume while reducing the average inter-arrival intervals. This theoretical characterization provides a statistical basis for the hybrid detection logic described in the experimental layout.

Overall, to support universal deployment without relying on vehicle-specific CAN IDs or payload semantics, we preprocess raw CAN logs into vehicle-agnostic timing and traffic-volume statistics and analyze them at two temporal resolutions: 0.01 s segments aggregated into a 1 s window (100 segments). In attack-free operation, these two statistics exhibit an approximately periodic structure, and are naturally coupled in an inverse manner: when the data-generation volume (packet count) increases, the mean inter-arrival time gap decreases, and vice versa. Consequently, any injection-based attack that introduces additional frames or perturbs message timing (e.g., DoS, fuzzing, replay, or spoofing-style injections) disrupts this periodic inverse coupling and becomes observable in the extracted statistics. Across various injection scenarios, misclassification can occur when injections are sparse and partially resemble benign periodic traffic; however, injected events are often discontinuous, and even a small amount of contamination within any sub-segment perturbs the window-level packet-count and inter-arrival-gap sequences. These localized deviations propagate into the extracted representations and become detectable either through the multi-resolution DWT detail coefficients (capturing time–frequency irregularities) or through the Pearson correlation dependency shift between volume and timing, thereby improving robustness and interpretability under cross-vehicle transfer settings.

## 4. Experimental Methodology

### Proposed System Architecture

**Figure 3:**
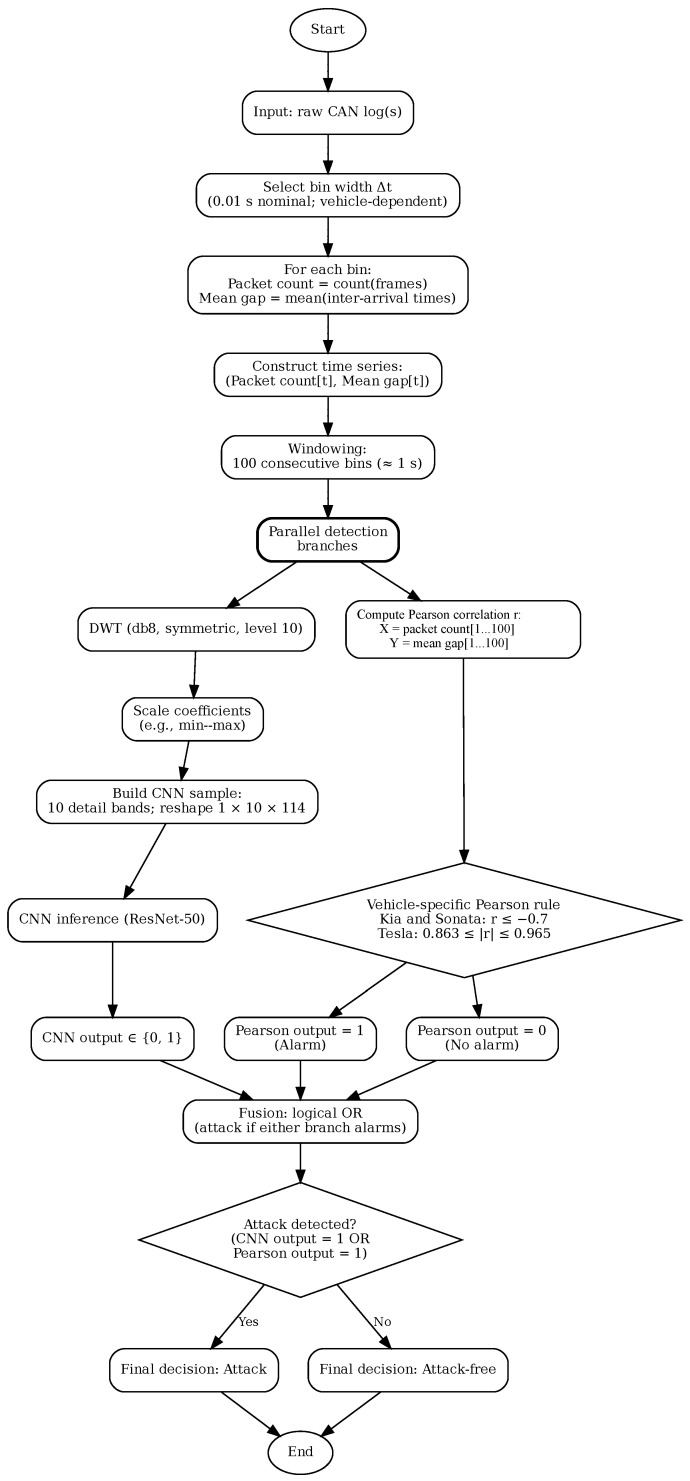
Block diagram of the proposed universal intrusion detection system (UIDS), illustrating the pipeline from CAN log segmentation to feature extraction, inference, and final decision.

The proposed architecture integrates deep learning feature extraction with statistical anomaly detection through a parallel processing pipeline. The specific procedural steps for data preparation, feature engineering, and hybrid decision-making are detailed in Algorithm 1 and the flowchart in Figure 3. All deep learning experiments in this study were conducted under a fixed model specification consisting of (i) a CNN detector implemented as a ResNet-50 backbone with a binary classification head (attack-free vs. attack), following the standard residual design commonly adopted for intrusion detection tasks [46] and using the exact layer-wise configuration and training setup reported in Table 2, and (ii) a transformer baseline implemented with the RoBERTa-base encoder architecture as defined in [47]. For the RoBERTa-based classifier, continuous time-series features (per-sample sequence length T=10, per-token feature dimension $d_{in}\;=\;114$) are provided through inputs_embeds by applying a learned linear projection 114→768 (RoBERTa hidden size), followed by the RoBERTa encoder stack; the final prediction is obtained by mean pooling over the last hidden states, applying dropout, and using a linear layer to produce two logits (attack-free vs. attack).

The UIDS evaluation employs a multi-vehicle dataset comprising DoS, fuzzing, and replay attacks. For the HCRL data, each scenario includes 300 intrusion events (3–5 s) to ensure adequate temporal resolution; DoS injections utilize the dominant ID=0×0000 at 0.3 ms periodicity, while fuzzing employs random frames every 0.5 ms. Replay attacks are generated by re-injecting previously captured benign CAN frames following the same injection procedure. For the LISA high-frequency setting, intrusions are injected at higher rates as specified by the LISA logs (Table 3). Robustness is validated via a transfer protocol: training on low-frequency HCRL data (Kia, Sonata) and testing against (i) an unseen HCRL vehicle and (ii) high-frequency LISA data (Kia, Tesla). Detailed traffic statistics are summarized in Table 3.

The operational workflow commences with the ingestion of raw CAN logs from multiple vehicle sources and, following Step 1 of Algorithm 1, partitions each log into discrete segments of width Δ to compute two timing-only statistics per segment *s*: the packet count $N_{\mathrm{pkt}}\left[s\right]$ and mean inter-arrival gap $\bar{\Delta t}\left[s\right]$, forming synchronized time-series sequences. Although a nominal Δ=9 ms is used in our baseline setting, to prevent vehicle-dependent manual tuning and preserve universality, we apply a vehicle-agnostic data-adaptability calibration: for each raw log, we evaluate a small candidate set of segment lengths, and for each candidate, we reuse the same segmentation and Pearson correlation computation in the pipeline to quantify benign stability. The segment length that maximizes this benign-stability score is then selected automatically, and the remainder of the hybrid IDS remains unchanged (wavelet features, ResNet inference, Pearson decision, OR fusion), ensuring robustness to heterogeneous traffic rates based on observed variability rather than vehicle identity.
**Algorithm 1:** Experimental workflow of the proposed universal intrusion detection system (UIDS)
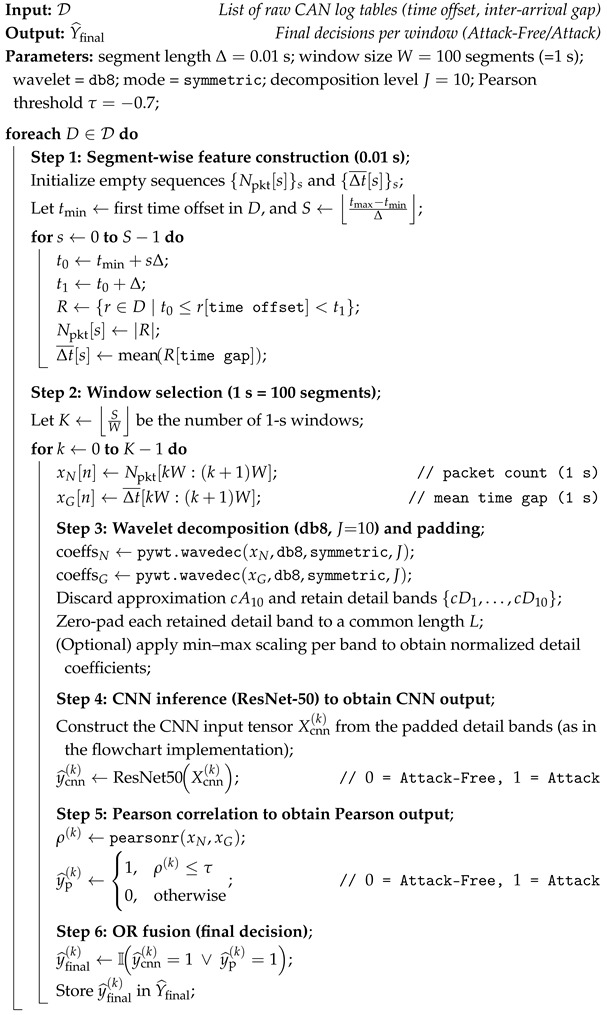


**Figure 4:**
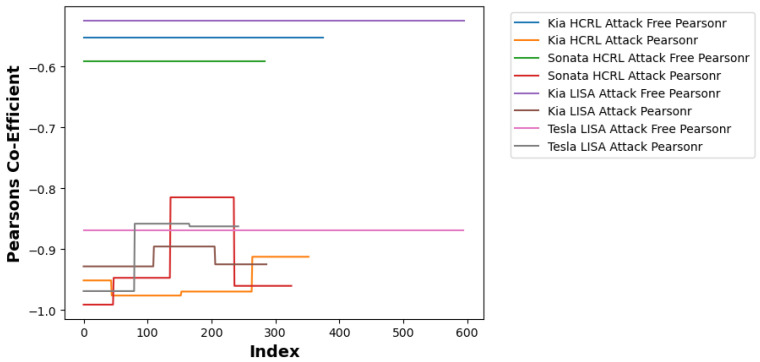
Pearson coefficient threshold.

Following feature construction, the system applies a windowing function to aggregate W=100 consecutive segments, corresponding to approximately 1 s of driving statistics. This windowed data is then processed through two parallel detection branches as defined in Step 2 of the algorithm:

- Deep Learning Branch (DWT and CNN): The sequences for packet counts and mean gaps are subjected to a 10-level DWT using the Daubechies 8 (db8) wavelet with symmetric boundary extension. As described in Step 3, we discard the approximation band $cA_{10}$ and retain the ten detail bands $\left\{cD_{1},\ldots ,cD_{10}\right\}$. These coefficients undergo zero-padding and min–max scaling to ensure a uniform 1×10×114 input tensor. Inference is performed using a ResNet-50 architecture, which utilizes 50 layers and shortcut connections to optimize gradient flow during binary classification.
- Statistical Branch (Pearson Correlation): Concurrently, the system computes the Pearson correlation coefficient (ρ) between the packet-count sequence and the mean time-gap sequence within each 1-s window. For the Tesla (LISA) traffic profile, a fixed single-threshold rule (ρ≤−0.7) (See Figure 4) produced unstable behavior due to normal traffic flow correlation variability. Therefore, this study applies a Tesla-specific stability-band rule defined on the absolute correlation: a window is classified as attack-free only when |ρ|∈[0.863,0.965], and is flagged as an attack otherwise. This band is empirically determined from Tesla attack-free calibration traces and is used to mitigate false alarms under sensor-driven EV traffic dynamics.

The final stage of the experimental layout involves Step 6 (Binary Voting), where the outputs from the CNN branch (${\hat{y}}_{\mathrm{cnn}}$) and the Pearson branch (${\hat{y}}_{p}$) are combined. The system employs a logical OR fusion mechanism: an “attack” decision is finalized if either branch generates an alarm signal. Conversely, the window is classified as attack-free only if both independent methodologies yield a null result. This hybrid approach enhances robustness by capturing both non-linear patterns through deep learning and linear statistical deviations through correlation analysis.

## 5. Results and Evaluation

The study utilizes two frequency injections: low-rate periodic injections from HCRL and high-frequency injections from LISA. The study aims to evaluate the performance of a machine learning algorithm trained on two different vehicle datasets, Sonata and Kia, using low-frequency periodic injection during the training phase. The algorithm’s effectiveness was tested in four scenarios: Sonata to Sonata low-frequency periodic injection, Sonata to Kia low-frequency periodic injection, Sonata to Kia high-frequency injection, and Sonata to Tesla high-frequency injection. The same approach was applied to the Kia-trained algorithm.

To evaluate detection performance under cross-vehicle and cross-frequency shifts, we report standard binary-classification metrics computed from the confusion matrix entries: true positive (TP), true negative (TN), false positive (FP), and false negative (FN), where the positive class denotes attack. Accuracy, precision, recall, and $F_{1}$ score are defined as

(4) $$\mathrm{Acc}=\frac{\mathrm{TP}+\mathrm{TN}}{\mathrm{TP}+\mathrm{TN}+\mathrm{FP}+\mathrm{FN}},$$

(5) $$\mathrm{Prec}=\frac{\mathrm{TP}}{\mathrm{TP}+\mathrm{FP}},$$

(6) $$\mathrm{Rec}=\frac{\mathrm{TP}}{\mathrm{TP}+\mathrm{FN}},$$

(7) $$F_{1}=\frac{2\;\mathrm{Prec}\;\mathrm{Rec}}{\mathrm{Prec}+\mathrm{Rec}}.$$

In addition, we report the area under the ROC curve (AUC), where $\mathrm{TPR}=\frac{\mathrm{TP}}{\mathrm{TP}+\mathrm{FN}}$ and $\mathrm{FPR}=\frac{\mathrm{FP}}{\mathrm{FP}+\mathrm{TN}}$; AUC summarizes the ROC trade-off over decision thresholds and is computed as

(8) $$\mathrm{AUC}=\int_{0}^{1}\mathrm{TPR}\left(\mathrm{FPR}\right)\;d\left(\mathrm{FPR}\right).$$

All metrics are computed per test scenario using the corresponding predicted labels and ground truth.

**Figure 5:**
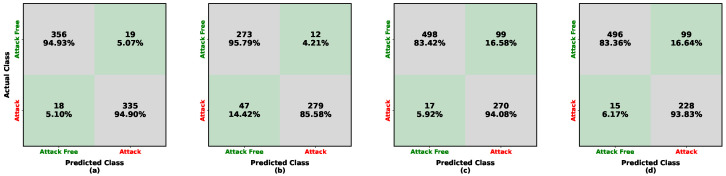
Confusion matrices illustrating the performance of the ResNet-50 detector trained on the Kia (HCRL) dataset. Each cell reports the number of samples and the corresponding row-normalized percentage. Color shading encodes the percentage magnitude: diagonal (green) cells indicate correct classifications, whereas off-diagonal (gray) cells indicate misclassifications. The subfigures represent: (**a**) in-domain testing on Kia (HCRL); (**b**) cross-vehicle testing on Sonata (HCRL); (**c**) cross-frequency testing on Kia (LISA); and (**d**) cross-vehicle and cross-frequency testing on Tesla (LISA).

**Figure 6:**
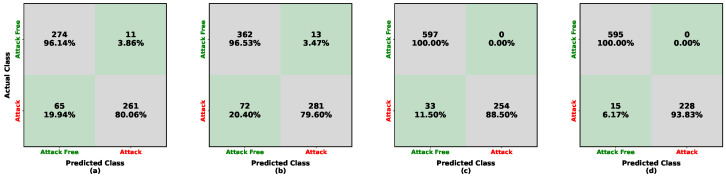
Confusion matrices illustrating the performance of the ResNet-50 detector when trained on the Sonata (HCRL) dataset. Each cell reports the number of samples and the corresponding row-normalized percentage. Color shading encodes the percentage magnitude: diagonal (green) cells indicate correct classifications, whereas off-diagonal (gray) cells indicate misclassifications. The subfigures represent: (**a**) in-domain test on Sonata (HCRL); (**b**) cross-vehicle test on Kia (HCRL); (**c**) cross-frequency test on Kia (LISA); and (**d**) cross-vehicle and frequency test on Tesla (LISA).

The performance evaluation of the deep learning model is shown in Figure 5 and Figure 6 and in Table 4. These results characterize the model’s attack-detection capability under multiple train–test transfer scenarios across heterogeneous vehicles. When trained on Kia data, the model achieves approximately 96% accuracy on attack-free samples for both Kia and Sonata under low-frequency periodic injection, as shown in Figure 5a,b. However, the accuracy for attack detection decreases, reaching approximately 95% on Kia and 86% on Sonata. Under high-frequency injection evaluated on the LISA datasets, the attack-free detection accuracy further declines to approximately 84% for both Kia and Tesla (Figure 5c,d), while attack samples are detected at approximately 94% for both vehicles.

Table 4 provides a consolidated comparison of ResNet-50 and a transformer baseline (RoBERTa) under identical transfer configurations. When trained on Kia (HCRL), ResNet-50 attains an F1/accuracy/AUC of approximately 90–91% on the cross-vehicle Sonata (HCRL) test and approximately 95% on the in-domain Kia (HCRL) test. Under high-frequency injection on the LISA datasets, performance decreases to an F1/accuracy of approximately 87% with an AUC of approximately 89% on both Kia (LISA) and Tesla (LISA), indicating sensitivity to cross-domain distribution shifts. In contrast, RoBERTa matches or exceeds ResNet-50 under low-frequency periodic injection, maintaining approximately 95% across F1/accuracy/AUC for both Sonata (HCRL) and Kia (HCRL), but degrades substantially under high-frequency injection on the LISA datasets, dropping to approximately 73% across F1/accuracy/AUC on Kia (LISA) and to approximately 76–83% on Tesla (LISA).

In comparison, the Sonata-trained model shows stronger generalization under low-frequency periodic injection, as illustrated in Figure 6a,b. Table 4 confirms that, when trained on Sonata (HCRL), ResNet-50 achieves approximately 88% F1/accuracy/AUC on the Sonata (HCRL) test set and approximately 88–89% on the Kia (HCRL) test set. Under high-frequency injection on the LISA datasets, the performance increases substantially, reaching approximately 94–96% on Kia (LISA) and approximately 97–99% on Tesla (LISA), indicating improved robustness for cross-domain high-frequency injection.

Table 4 also reports a transformer baseline (RoBERTa) under the same transfer configurations. RoBERTa remains competitive under low-frequency periodic injection, maintaining approximately 95% across F1/accuracy/AUC on both Sonata (HCRL) and Kia (HCRL). However, under high-frequency injection on the LISA datasets, RoBERTa degrades markedly, dropping to approximately 70–73% on Kia (LISA) and approximately 76–83% on Tesla (LISA). Overall, the Sonata-trained ResNet-50 consistently exhibits stronger cross-vehicle robustness than the transformer baseline under high-frequency injection, motivating the subsequent hybrid IDS design that combines the deep learning detector with a complementary statistical component.

**Figure 7:**
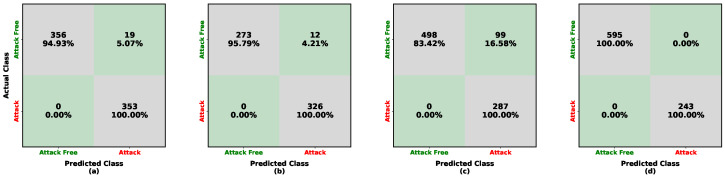
Confusion matrices illustrating the performance of the hybrid approach for UIDS when trained on the Kia (HCRL) dataset. Each cell reports the number of samples and the corresponding row-normalized percentage. Color shading encodes the percentage magnitude: diagonal (green) cells indicate correct classifications, whereas off-diagonal (gray) cells indicate misclassifications. The subfigures represent: (**a**) in-domain test on Kia (HCRL); (**b**) cross-vehicle test on Sonata (HCRL); (**c**) cross-frequency test on Kia (LISA); and (**d**) cross-vehicle and frequency test on Tesla (LISA).

**Figure 8:**
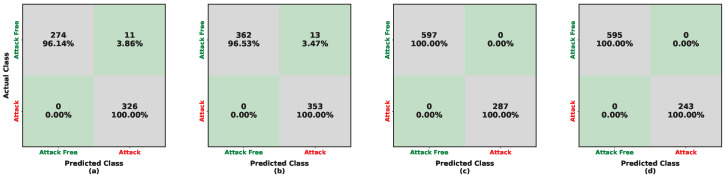
Confusion matrices illustrating the performance of the hybrid approach for UIDS when trained on the Sonata (HCRL) dataset. Each cell reports the number of samples and the corresponding row-normalized percentage. Color shading encodes the percentage magnitude: diagonal (green) cells indicate correct classifications, whereas off-diagonal (gray) cells indicate misclassifications. The subfigures represent: (**a**) in-domain test on Kia (HCRL); (**b**) cross-vehicle test on Sonata (HCRL); (**c**) cross-frequency test on Kia (LISA); and (**d**) cross-vehicle and frequency test on Tesla (LISA).

A hybrid IDS was developed by combining a rule-based IDS (Pearson correlation coefficients) with a deep learning model. The performance evaluation of the hybrid IDS is shown in Figure 7 and Figure 8 and in Table 5. For low-frequency injection training with Kia, as shown in Figure 7a,b, the detection accuracy for attack-free samples from Kia and Sonata is approximately 96%, while attack samples are detected with 100% accuracy. For high-frequency injection, as shown in Figure 7c, attack-free Kia samples are detected at approximately 84%, while attack samples are detected at 100%. The updated Tesla evaluation in Figure 7d shows perfect separation between attack-free and attack samples, achieving 100% accuracy on Tesla (LISA). Consistent with Table 5, the model trained on Kia achieves 98% and 97% accuracy for low-frequency periodic injection (Sonata and Kia tests, respectively), and 90% (Kia LISA) and 100% (Tesla LISA) accuracy for high-frequency injection.

For low-frequency injection training with Sonata, as shown in Figure 8a,b, the detection accuracy for attack-free samples from Sonata and Kia is approximately 97%, while attack samples are detected with 100% accuracy. For high-frequency injection, as shown in Figure 8c, both attack-free and attack samples for Kia are detected at 100%. The updated Tesla evaluation in Figure 8d similarly achieves 100% accuracy on Tesla (LISA), indicating that the hybrid decision logic remains effective under EV traffic dynamics after applying the Tesla-specific correlation stability range. As reported in Table 5, the model trained on Sonata achieves 98% accuracy for low-frequency periodic injection (Sonata and Kia tests) and 100% accuracy for high-frequency injection on both Kia (LISA) and Tesla (LISA). When comparing our results with other studies, the overall accuracy is competitive with conventional IDS and UIDS baselines reported in Table 6.

## 6. Discussion

The observed error modes are primarily governed by the underlying injection frequency. Low-frequency periodic injections may be missed because their sparse volume emulates the characteristics of attack-free data flows. Conversely, high-frequency replay patterns can induce false positives within attack-free windows when they share statistical similarities with normal traffic. This frequency-driven domain shift explains the performance of the Transformer baseline (RoBERTa). RoBERTa succeeds in the mechanical-vehicle HCRL scenario because the Kia and Sonata datasets share identical injection frequencies, allowing self-attention to capture stable temporal patterns. However, this performance degrades when transferring to LISA datasets with different injection periodicities. By converting traffic statistics into image-like 2D multi-resolution tensors, the CNN-based detector (ResNet-50) utilizes spatial inductive biases to maintain invariance across these frequency shifts. Because Transformers lack this specialized spatial bias for 2D feature maps, they are treated as evaluative baselines rather than practical solutions for universalized detection.

The Pearson correlation branch is intentionally retained as an interpretable secondary detector because it achieves high separation under fixed-frequency and stable traffic conditions where malicious periodicity produces consistent linear deviations between packet-count and mean-gap signals. However, relying solely on a fixed threshold is a vulnerability as an adaptive adversary could perturb injection timing to mimic attack-free correlation patterns, so Pearson is coupled with a ResNet-50 detector capable of capturing higher-order, non-linear patterns that correlation alone cannot represent. The final decision is reached via a binary voting mechanism using a logical OR operation where a window is classified as an attack if either the deep learning branch or the Pearson branch raises an alarm, and is labeled attack-free only when both branches agree on the absence of malicious behavior. This fusion strategy explicitly improves resilience against threshold bypass under frequency variation while preserving the interpretability and low-cost advantages of the correlation rule. For high-variability traffic, such as electric vehicle dynamics, the Pearson branch is further stabilized through traffic-profile-aware calibration utilizing segment-length selection and stability-window bounds to minimize false alarms while providing complementary evidence to the fused decision.

Table 6 demonstrates that while many high-performing IDS studies achieve strong results, they often operate under constrained assumptions such as fixed protocols or limited transfer conditions. Specifically, the WINDS method—a wavelet-based statistical approach—attains high accuracy when evaluated on the Kia platform, indicating that frequency-centric handcrafted pipelines can be effective in a single-vehicle environment. However, under strict cross-vehicle transfer to the Sonata, the WINDS method fails completely, illustrating that rule-dependent segmentation and vehicle-specific traffic regularities do not reliably generalize to unseen platforms. This gap justifies our hybrid design, which combines a vehicle-agnostic, multi-resolution image-structured representation learned by a ResNet-50 with a lightweight Pearson correlation branch. These components are fused through an OR-based voting logic, where a window is classified as an attack if either branch raises an alarm. As summarized in Table 6, the proposed model sustains strong performance across heterogeneous vehicles and varying injection periodicities while maintaining interpretability through its secondary statistical branch.

## 7. Conclusions

This study addressed the lack of portability in CAN traffic distributions, which causes significant model degradation during cross-vehicle and frequency shifts. To achieve universality, we proposed a UIDS that bypasses brittle identifier and payload dependencies, relying instead on vehicle-agnostic timing and traffic-volume statistics. These features are transformed into a multi-resolution wavelet representation and processed via a parallel pipeline of ResNet-50 and Pearson correlation, fused through a logical OR decision rule. Evaluations across Kia, Sonata, and Tesla platforms demonstrate high detection accuracy and resilience against domain shifts. While effective, future work should address the current limitation of data accessibility across diverse vehicle manufacturer architectures and further reduce computational overhead to meet stringent in-vehicle ECU hardware constraints.

## Figures and Tables

**Table 1:** Comparative analysis of vehicle CAN data characteristics across different platforms and data collection years.

Vehicle/Dataset	Data Collection Year	Number of CAN IDs	Data Length (s)	Average Time Gap (s)	Chunk Size (ms)
Kia (LISA)	2022	45	2085	0.4772	10
Kia (HCRL)	–	45	2085	0.4772	9
Sonata (HCRL)	–	27	1943	0.5132	9
Tesla (LISA)	2021	69	3126	0.3195	6.5
Tesla (LISA)	2022	121	1652	0.6095	10.0
Tesla (LISA)	2024	267	2871	0.3476	8.0
Tesla (LISA)	2025	156	2472	0.4032	8.0

**Table 2:** ResNet-50 architecture and training hyperparameters specification.

Category/Item	Specification
Architecture	
Network	ResNet-50 (custom implementation)
Input channels/classes	$C_{\mathrm{in}}=1$, #classes =2
Stem conv	7×7, 64 filters, stride 2, padding 3
Stem norm/act	BatchNorm2d + ReLU
Stem pooling	MaxPool 3×3, stride 2, padding 1
Block type	Bottleneck block, expansion factor =4
Bottleneck structure	1×1 (reduce) → 3×3 (stride *s*) → 1×1 (expand)
Normalization	BatchNorm2d after each conv
Activation	ReLU (after BN; and after residual addition)
Stage depths (layers)	Layer1: 1 block @ 64|Layer2: 4 blocks @ 128|Layer3: 6 blocks @ 256|Layer4: 3 blocks @ 512
Stage strides	Layer1: s=1; Layer2: s=2; Layer3: s=2; Layer4: s=2
Identity downsample	1×1 conv with stride *s* + BN when s≠1 or channels mismatch
Pooling head	AdaptiveAvgPool2d(1,1)
Classifier head	Linear(512×4→2)
Output	2 logits (benign vs. attack)
Hyperparameters	
Loss	CrossEntropyLoss (reduction = sum)
Optimizer	Adam
Learning rate (η)	learning_rate (report numeric value)
LR scheduler	ExponentialLR, γ=0.1 (step each epoch)
Early stopping	Validation loss, patience =10, delta =0

**Table 3:** Statistical profile of the multi-vehicle CAN bus dataset for cross-platform and frequency-invariant UIDS validation, detailing traffic volume and injection periodicity across heterogeneous sources (HCRL and LISA).

Vehicle	Source	Type	Total Data	Injection Frequency Approximately (s)
Kia	HCRL	Attack-Free	665,726	2092
		DoS	36,805	1800
		Fuzz	342,355	4800
		Replay	363,145	1800
	LISA	Attack-Free	939,237	2094
		DoS	1,877,487	2300
		Fuzz	1,877,487	2300
		Replay	1,877,487	2300
Sonata	HCRL	Attack-Free	431,134	1956
		DoS	56,829	1200
		Fuzz	382,577	1200
		Replay	517,257	1200
Tesla	LISA	Attack-Free	599,999	3124
		DoS	1,198,394	6248
		Fuzz	1,198,394	6247
		Replay	1,198,394	6247

**Table 4:** Performance metrics of CNN (Resnet-50) and Transformer (RoBERTa) architectures for UIDS across different vehicle datasets and injection frequencies.

Train Vehicle/Model	Low-Frequency Periodic Injection						High-Frequency Injection					
	Sonata Test (HCRL)			Kia Test (HCRL)			Kia Test (LISA)			Tesla Test (LISA)		
	F1	Acc	AUC	F1	Acc	AUC	F1	Acc	AUC	F1	Acc	AUC
Kia (HCRL)—ResNet-50	0.90	0.90	0.91	0.95	0.95	0.95	0.87	0.87	0.89	0.87	0.87	0.89
Kia (HCRL)—RoBERTa	0.95	0.95	0.95	0.95	0.95	0.95	0.73	0.73	0.73	0.78	0.76	0.83
Sonata (HCRL)—ResNet-50	0.88	0.88	0.88	0.89	0.88	0.88	0.96	0.96	0.94	0.99	0.98	0.97
Sonata (HCRL)—RoBERTa	0.95	0.95	0.95	0.95	0.95	0.95	0.73	0.70	0.72	0.78	0.76	0.83

**Table 5:** Performance metrics of the hybrid approach (ResNet-50 + Pearson) for UIDS across different vehicle datasets for low and high-frequency injection attacks.

Train Vehicle	Low-Frequency Periodic Injection						High-Frequency Injection					
	Sonata Test (HCRL)			Kia Test (HCRL)			Kia Test (LISA)			Tesla Test (LISA)		
	F1 Score	Acc	AUC	F1 Score	Acc	AUC	F1 Score	Acc	AUC	F1 Score	Acc	AUC
Kia (HCRL)	0.98	0.98	0.98	0.97	0.97	0.97	0.90	0.90	0.92	1.00	1.00	1.00
Sonata (HCRL)	0.98	0.98	0.98	0.98	0.98	0.98	1.00	1.00	1.00	1.00	1.00	1.00

**Table 6:** Comparative analysis of IDS techniques: performance and training methodologies.

Approaches	Author	Re-Implement	Features	Datasets	Algorithm	Training Approach	Split Ratio	Performance	Limitations
Conventional	Lo et al. [27]	No	Full Data	HCRL	CNN, LSTM	Supervised	67/33	100%	High computation
	Mansourian [25]	No	Payload	HCRL	LSTM-GNB	Hybrid	20/80	100%	High computation
	Jedh et al. [26]	No	CAN ID	Authors	Pearson, LSTM	Hybrid	70/30	98.45%	No payload analysis
	Yu et al. [2]	No	Time gap	HCRL	TCE-IDS (CNN)	Supervised	70/30	99%	Rule-dependent
	Xun et al. [24]	No	Voltage	Authors	KNN	Supervised	80/20	97%	Hardware dependent
Universal	Bozdal et al. [18]	Yes	Frequency	HCRL, LISA	WINDS (CWT)	Statistical	Segmented	97% (Kia)	Rule-dependent
	Novikova et al. [17]	No	Signals	2.7B Frames	Autoencoder	Unsupervised	Not Specified	99%	Offline focus
	Islam et al. [19]	Yes	CAN ID, Time Gap, Hamming Distance	LISA	CNN	Supervised	80/20	99%	High-freq focus
Proposed			Entropy, Time Gap	HCRL, LISA	CNN + Pearson	Supervised	N/A	97–100%	Limited Dataset

## Data Availability

The data supporting the findings of this study are publicly available. The primary dataset, titled “UIDS-CAN: A Multi-Vehicle CAN Intrusion Detection Dataset”, has been archived on IEEE DataPort and is accessible at: https://ieee-dataport.org/documents/uids-can-multi-vehicle-can-intrusion-detection-dataset (accessed on 21 February 2026). The dataset includes multi-vehicle CAN bus traffic collected under normal driving conditions and controlled cyber-attack scenarios (DoS, fuzzing, and replay) across heterogeneous platforms, including mechanical and electronic vehicles. Additionally, this study utilizes the HCRL dataset as a supplementary benchmark for low-frequency periodic injection analysis. No personally identifiable information is included, and all data were collected in compliance with applicable ethical and privacy guidelines.
